# Current State of Platelet-rich Plasma in the Treatment of Rheumatic Disease: A Retrospective Review of the Literature

**DOI:** 10.2174/1573397119666230420112017

**Published:** 2023-08-03

**Authors:** Adam Jacobs, Omar Elghawy, Diego Lugo Baruqui, Ahmed Aly Elghawy

**Affiliations:** 1 Mount Sinai Medical Center, Miami Beach, FL, United States;; 2 University of Virginia School of Medicine, Charlottesville, VA, United States;; 3 Department of Rheumatologic and Immunologic Disease, Cleveland Clinic, Cleveland, OH, United States

**Keywords:** Platelet-rich plasma, rheumatic disease, rheumatoid arthritis, vasculitis, Sjogren’s syndrome, crystalline-arthropathy, osteoarthritis

## Abstract

**Introduction:**

Rheumatic diseases are a spectrum of autoimmune or inflammatory diseases that cause damage to the musculoskeletal system as well as vital organs, such as the heart, lungs, kidneys, and central nervous system.

**Methods:**

The study of rheumatic disease has made great progress in the understanding and management of these conditions in the last few decades using disease-modifying antirheumatic drugs and synthesized biological immunomodulating therapies. However, one potential treatment that has not been well investigated in rheumatic disease is platelet-rich plasma (PRP). PRP is proposed to facilitate the healing of injured tendons and ligaments through a variety of mechanisms, including mitogenesis, angiogenesis and macrophage activation *via* cytokine release, although its exact mechanism is unclear.

**Results:**

There has been a great deal of work in determining the exact preparation method and composition of PRP for regenerative purposes in orthopedic surgery, sports medicine, dentistry, cardiac surgery, pediatric surgery, gynecology, urology, plastic surgery, ophthalmology, and dermatology. Despite this, there is a paucity of research on the impact of PRP on rheumatic disease.

**Conclusion:**

This study aims to summarize and evaluate the current research concerning the use of PRP in rheumatic disease.

## 1 INTRODUCTION

Rheumatic diseases (RD) are a spectrum of autoimmune conditions that can cause damage to the musculoskeletal system as well as other vital organs, such as the heart, lungs, kidneys, and central nervous system. Rheumatic diseases are becoming increasingly common due to longer life expectancy and an aging general population, with a prevalence of about 1 in 4 adults in the United States [1]. The Center for Disease Control has recently predicted that their prevalence will likely continue to increase by 49% by 2040 [2].

There have been significant advances in the study and understanding of pathophysiologic mechanisms and therapeutic interventions involving RD over the last few years. Through the growth of the medical armamentarium involving the use of glucocorticoids, disease-modifying antirheumatic drugs (DMARDs) and newer synthesized biological immunomodulating agents, physicians are now able to positively impact the prognosis, quality of life, and functional capacity of these patients [3-5]. However, despite the advancement of available therapies, some limitations persist, and many patients are often not suitable for long-term management due to lack of lasting efficacy, side effects, cost, and lack of insurance coverage, all of which can contribute to discontinuation and suboptimal outcomes [6, 7].

**Fig. (1):**
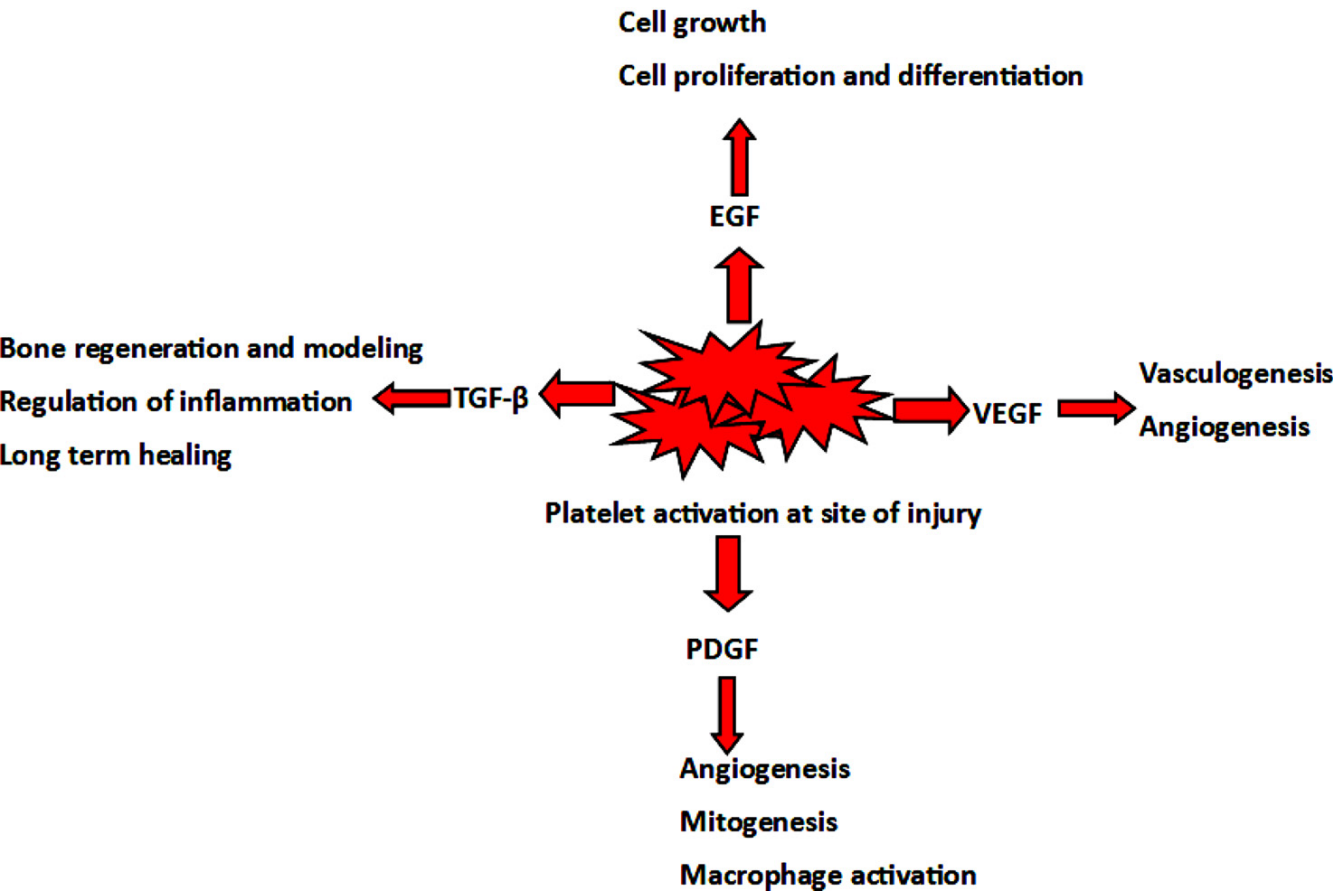
Diagram demonstrating many of the mechanisms by which platelet rich plasma decreases inflammation.

One potential treatment option that has not been well investigated or FDA-approved in RD is the use of platelet-rich plasma (PRP). PRP is an autologous blood product composed of a processed liquid fraction of peripheral blood with a platelet concentration above the baseline [8]. The exact mechanisms by which PRP exerts its clinical benefits are unclear. It is believed to stimulate the expression of anti-inflammatory molecules in high concentrations. These molecules include growth factors, such as vascular endothelial growth factor (VEGF), transforming growth factor-β (TGF- β), epidermal growth factor (EGF), fibroblast growth factor (FGF), and platelet-derived growth factor (PDGF) (Fig. **1**) [9].

Significant research has been conducted to determine the most effective composition and PRP concentration needed to achieve the greatest reduction in the inflammatory response. The impact of the final PRP volume, platelet, EGF, VEGF, leukocyte concentrations, and platelet activation markers has been extensively studied in the literature [10]. However, many studies still use different preparations, which may influence results.

The potential benefits of PRP use at the cellular/genetic level are demonstrated in several studies, including one by Tong *et al*. in 2017 [11]. The study showed that PRP led to a significant reduction in proinflammatory cytokines and reversed the negative effects of lipopolysaccharide, which was shown to simulate the conditions of rheumatoid arthritis (RA).

Recently, PRP has shown promising results as an immunomodulatory agent in cell and animal models [12-16]. Given these findings, PRP has been investigated in several areas of the medical field, including regenerative medicine in orthopedic surgery, sports medicine, dentistry, cardiac surgery, pediatric surgery, gynecology, urology, plastic surgery, ophthalmology, and dermatology [17, 18]. Nevertheless, the results of randomized clinical trials have been mixed [19-24]. This retrospective narrative review aims to summarize results and provide a state-of-the-art analysis of the clinical benefits of PRP use and its possible side effects in a variety of RD.

## 2 METHODS

A structured literature search was performed *via* PubMed using keywords “Rheumatoid arthritis”, “Inflammatory Arthritis”, “Platelet”, “Platelet Rich Plasma”, “Psoriatic Arthritis”, “ Psoriasis”, “ Reactive Arthritis”, “Gout”, “Vasculitis”, “Giant Cell Arteritis”, “Polyangiitis”, “Inflammatory Myopathy”, “Myositis”, “Polymyositis”, “Dermatomyositis”, “Lupus”, “ Systemic Lupus Erythematosus”, “ Discoid Lupus”, “Ankylosing Spondylitis”, “Scleroderma”, “Crest Syndrome”, “Sarcoid”, “Sarcoidosis”, “IgG4-Related Disease”, along with Boolean operators “AND”, “OR”, and “NOT”. The search was limited to English language articles within the last 5 years as we found the most relevant papers, more specifically human studies and case reports, which had been published within this time period. This search strategy identified 5320 articles. Studies were excluded from this study if they were bibliographies, comments, editorials, interviews, lectures, legal cases, legislation, letters, news, newspaper articles, patient education handouts, popular works, congress, consensus development conferences, practice guidelines, or reviews. Studies were also excluded if they did not include primary data relating to the use of PRP in RD defined by the American College of Rheumatology. Fourteen articles met the inclusion criteria and were, therefore, included in this review (Table **1**). References of included articles were examined to find additional articles. A detailed study selection flow diagram is given in Fig. (**2**).

## 3 RESULTS

### 3.1 Rheumatoid Arthritis

A 2021 open-label randomized controlled trial (RCT) performed by Saif *et al*. analyzed the use of PRP *versus* intra-articular steroid injection in 60 patients with RA who had low disease activity but persistent pain or inflammation in 1-2 medium-sized joints despite the use of non-biologic DMARDs [25]. They assessed scores on the visual analog scale (VAS) for pain, swelling, and tenderness in addition to the Health Assessment Questionnaire (HAQ) Disability Index. Other outcomes evaluated included Disease Activity Score 28 (DAS 28) and serum levels of IL-1β and TNF-ɑ. The first group included 30 patients who received monthly injections of 3-4 ml of PRP (with a reported average platelet count of 400,000 - 1,000,000/ml) over 3 months. Treated joints included 24 wrists, 10 elbows, and 12 ankles. The second group also included 30 patients who received a single injection of 40 mg of triamcinolone acetonide. Treated joints in this group included 22 wrists, 10 elbows, and 11 ankles. Both groups showed improvement in the VAS as well as in the HAQ Disability Index at 3 months.

However, only the PRP group showed sustained improvement of symptoms at 6 months (*p*-value < 0.0001). The same results were observed in the DAS 28 and post-treatment serum levels of IL-1β and TNF-ɑ. IL-1β levels in the PRP group decreased progressively from 8.76 to 4.74 at 6 months (*p*-value < 0.0001), whereas in the steroid group, there was no significant difference from baseline at 6 months. Similarly, the TNF-ɑ levels in the PRP group also progressively decreased from 31.74 to 18.3 at 6 months (*p*-value = 0.003); yet again, there was no significant difference in the steroid-treated group at 6 months. No significant difference in adverse effects was reported.

Moreover, a case series by Shively *et al*. in 2021 reported the outcomes of three patients who were treated with 0.5 ml of intra-articular PRP and 1.5 ml of peri-articular PRP [26]. The first patient received treatment in the right fourth and fifth metacarpophalangeal (MCP) joints; the second patient was injected in the right third proximal interphalangeal (PIP) joint; the third patient was treated in the right third MCP and right fifth PIP joint. The Patient Activity Scale II (PAS-II) was used to assess disease severity on the day of injection and again at 1, 3, and 6 months. All three patients reported improvement on the PAS-II regarding pain and functional capacity; no significant adverse effects were noted.

Another case series, reported by Badsha *et al*. in 2020, assessed the effects of intra-articular leukocyte-poor PRP injection in four patients with concomitant RA and osteoarthritis who had persistent pain and inflammation despite steroid injections and DMARD use [27]. The first patient received a 2 ml injection of PRP in the right wrist and showed significant improvement in the VAS and the DAS 28. Additionally, a lower grade of synovitis at 6-week and 12-week follow-ups was noted. The second patient was administered 4 ml injections of PRP in both knees and reported significant improvement in the VAS and DAS 28 scores at 12 weeks. The third patient received a 4 ml PRP injection in both knees. She had no significant improvement in the VAS or DAS 28 scores at 8 weeks. However, her degree of synovial hypertrophy improved from grade 2 to grade 1. The fourth and final patient was treated with a 4 ml PRP injection in both knees. At 4 weeks, she had a notable improvement in the VAS of the right knee but not on the left. A second injection was performed, and 4 weeks later, she showed significant improvement in her left knee. Furthermore, these findings were found to be sustained at 1 year, and her degree of synovial hypertrophy improved from grade 2 to grade 1. The study noted no adverse effects during the mean follow-up period of 5 months.

### 3.2 Systemic Sclerosis

A small case series published in 2017 by Virzì *et al*. evaluated six patients with cutaneous systemic sclerosis who were not taking DMARDS. They were administered PRP and lipoaspirates (derived from autologous adipose tissue) into the left and right perioral and malar areas. The goal of therapy was to improve facial skin elasticity, vascularization, and functionality [28]. PRP was injected first, followed by lipoaspirate injection 15 minutes later, along with 500 mg of methylprednisolone (to reduce local post-operative edema). Results demonstrated a significant improvement in facial morpho-functionality, skin elasticity, and capillary density in the maxillofacial region, measured by videodermatoscope at 3 months follow-up.

### 3.3 Sjogren’s Syndrome

A randomized prospective study by Avila *et al*. analyzed 30 patients with primary Sjogren’s syndrome (SS) who were administered ocular PRP plus hyaluronic acid artificial tears *versus* artificial tears alone for the management of severe xerophthalmia [29]. Patients had to meet at least 2 of the American College of Rheumatology criteria for SS. Patients in the intervention group received PRP injections on days 0, 30, 60, and 90, along with daily artificial tears. Significant improvement in lacrimal volume was noted by 90 days in the patients receiving PRP. However, the control group (only treated with artificial tears daily) had no significant changes in lacrimal volume at 90 days compared to baseline (*p*-value <0.002). Tear break-up time improved significantly in the PRP group as well, while there was no significant change in the control group (*p*-value 0.0055). Similar findings were noted regarding improvement in ocular surface staining. The patients that received PRP had a significant improvement in the Ocular Surface Disease Index compared to the control group (34 ± 4.0 *vs*. 55 ± 2.5, *p*-value <0.001).

Additionally, Bottegoni *et al*. published a pilot study on 18 patients with primary SS and symptomatic knee osteoarthritis who were given a series of three PRP injections fourteen days apart [30]. They were followed on days 7, 30, and 90. Nearly 78% of these patients considered these injections satisfactory in improving pain based on the VAS and improving knee functionality based on patient/investigator global assessment.

### 3.4 Vasculitis

From an integumentary perspective, PRP has been studied considering its benefits in wound healing in patients with cutaneous vasculitis. A case report by Yi *et al*. discussed a 46-year-old female with non-healing lower extremity ulcers with black eschars secondary to cutaneous leukocytoclastic vasculitis [31]. Following topical administration of PRP gel, the wounds healed over the course of 1 month, with marked wound size reduction and new tissue formation. Similar findings were noted in a pilot study in 2016 by Sriram *et al*. [32], which included 20 patients with biopsy-proven vasculitic ulcers that did not heal despite 3 months of high-dose steroids and/or cyclophosphamide/mycophenolate mofetil treatment. Seven patients had systemic lupus erythematosus (SLE), 3 had RA, 3 had SS, 2 had dermatomyositis, and 2 had mixed connective tissue disease (MCTD). The remaining 3 patients had vasculitis that was not classified. A total of 10% calcium chloride was added to PRP in a 1:10 (PRP:CaCl) ratio five minutes before topical administration on the ulcer, followed by occlusive dressing. This was repeated weekly until complete healing was noted; immunosuppressive therapy was not halted during this study. All 20 patients exhibited complete healing of ulcers within 6 weeks. MCTD patients displayed the quickest time to heal at 3.5 weeks, while the other patients noted healing at around 4-5 weeks.

In a small study conducted in December 2021, Huber *et al*. examined oral ulcer healing in patients with Behcet’s disease (BD) not on DMARDs, using PRP as adjunctive therapy to colchicine 0.5 mg per day and prednisone 10 mg per day [33]. Eight patients were included, but only 6 completed the follow-up period. Autologous leukocyte-poor PRP was administered subcutaneously over the course of six months. The first six applications were given every fifteen days, followed by three applications every 30 days. Patients were followed up every three months for up to twelve months. Results showed decreased ulcer closure time and decreased frequency of ulcers during the first three months, with stabilization during the rest of the application period. There was a subsequent increase in the number of ulcers as well as closure time after the application period ended. In addition, there was an increase in T regulatory cells and a decrease in activated NK69 cells, though a rebound effect into a pro-inflammatory state after completion of therapy was observed.

### 3.5 Sarcoidosis

There were two case reports in 2017 and 2020 in which PRP use was called into question regarding the development of sarcoid granulomas or cutaneous sarcoidosis in regions of PRP injection. The first case report in 2017 by Serizawa *et al*. [34] reported a 68-year-old female diagnosed with sarcoidosis after developing multiple sarcoid granulomas in regions where PRP was injected to treat wrinkles. Treated regions were injected 12 times over 2 years. She presented with dim eyesight, uveitis with granulomatous keratic precipitates, and vitreous opacity consistent with ocular sarcoidosis. However, it is important to note that the patient had been previously treated with hyaluronic acid (HA) and botulinum toxin; thus, it was unclear whether PRP use was the causative factor for her sarcoid granulomas (as HA and botulinum injections consist of foreign material which has been previously documented to be related to sarcoid granuloma development). Regardless, it has been hypothesized that PRP injections could be involved in triggering granuloma formation.

The second case report in 2020 by Izhakoff *et al*. [35] reported a case of a 62-year-old female who developed cutaneous sarcoid lesions at PRP injection sites. In contrast to the prior case, she never received prior HA, botulinum toxin, or any other foreign material injection. The lesions developed 4 months after treatment of a right rotator cuff injury in addition to facial cosmetics. She was diagnosed with sarcoidosis 2 years later. The authors suspect a Koebnerization phenomenon led to the development of cutaneous sarcoidosis. However, it is unknown if the patient had undiagnosed subclinical sarcoidosis prior to the injections, as she did have a history of uveitis, which is a common clinical feature.

### 3.6 Crystalline Arthropathies

There is limited data regarding the benefits of PRP use in patients with crystalline arthropathies. Furthermore, there are several case reports of crystalline-arthropathy flares precipitated by PRP injections for osteoarthritis. A case report in 2017 by Dr. Jeffrey Tsai documented a 71-year-old male with tophaceous gout who developed acute left knee monoarthritis after receiving a left knee injection of PRP [36]. Joint aspiration noted a white cell count of 95,000 x 10 ^6^/L with monosodium urate crystals and a negative gram stain. Although there was uncertainty as to what type of injection the patient received, he admitted that he had his blood drawn and centrifuged before the injection, leading to the presumption that it was PRP. His symptoms resolved with an intra-articular corticosteroid injection, and it was believed that the pro-inflammatory cytokines and growth factors from PRP triggered his gout flare. Similarly, a case series by Schroeder *et al*. demonstrated pseudogout flares after PRP injections [37]. Three patients, ranging from 60 to 85 years old, received intra-articular or peri-tendinous PRP injections for knee osteoarthritis, with subsequent development of knee effusions. Joint aspirations yielded cloudy or blood-tinged synovial fluid with calcium pyrophosphate crystals and negative gram stain/cultures. Only one had a reported history of pseudogout before PRP treatment. Symptoms occurred within 5 days post PRP injection, and intra-articular steroid injections were used to resolve symptoms of the flares.

### 3.7 Discoid Lupus Erythematosus

We found one case report by Polster *et al*. [38] of a patient with scarring alopecia from discoid lupus erythematosus. The patient was treated with 3 doses of PRP to the area of scarring. Each dose was administered subcutaneously 3 months apart. Patients were found to have reduced scarring and improved hair growth *via* global photography.

## 4 DISCUSSION

The current literature review indicates that there is a paucity of evidence regarding the use of PRP in patients with RD, and larger clinical trials are needed to broaden our understanding of potential PRP benefits in these patients. The mentioned studies showed mixed results, and there appear to be variable reactions to PRP use depending on the patient population treated. For instance, patients with RA showed improvement in symptom severity and inflammatory marker levels after receiving PRP compared to intra-articular steroid injections, as measured by the DAS 28 and IL-1β and TNF-ɑ levels, respectively. Furthermore, this improvement was noted to be sustained 6 months after treatment. However, longer follow-up period data is needed to assess the longevity of such benefits.

On the other hand, potential adverse events were noted in patients with OA receiving PRP injections. While patients in these reports developed gout and pseudogout flares after PRP injections, the mechanisms remain unclear. However, it has been hypothesized that this could be secondary to the formation of radical oxygen species and activation of oxidative stress pathways after PRP utilization. Unfortunately, there have been no studies demonstrating the benefits of PRP use in patients with crystalline arthropathy. Moreover, another matter of debate is whether PRP use is associated with the development of sarcoid granulomas or cutaneous sarcoidosis in regions of PRP injection. Some of these patients received HA or Botulinum toxin as well, leading to uncertainty as to which substance could have triggered an abnormal immune activation and granuloma formation. Therefore, the exact mechanism by which these flares occurred is not well understood.

Additionally, PRP use was beneficial in boosting cutaneous wound healing in patients with various forms of vasculitis that were previously non-healing despite high doses of glucocorticoids or other immunosuppressive agents. Even patients with BD and oral ulcerations showed improvement after PRP use. Different formulations of PRP were used; however, longer follow-up data are needed to establish the frequency of wound recurrences. Interestingly, there appears to be a dermatologic benefit of PRP in patients with systemic sclerosis. However, the sample size was small, and the duration of follow-up remained insufficient to make statements about the long-term impact beyond 3 months. Also, PRP showed promising results in the treatment of SS, both in decreasing articular symptoms and increasing lacrimal production. However, the follow-up of these patients was limited to 3 months.

Information concerning the use of PRP in other RD is largely limited and insufficient to fully establish the role of PRP in managing these conditions. For example, we identified no significant data concerning the use of PRP in seronegative spondyloarthropathies, IgG4-related disease, or SLE. There are some data related to PRP use in non-inflammatory degenerative sacroiliitis, which is generally favorable. However, no papers were found in our time frame search addressing seronegative inflammatory sacroiliitis [39-46]. While there was no information concerning the use of PRP in psoriatic arthritis, there were 3 papers (2 reviews by Lin *et al*. and White *et al*. and one retrospective study by Kauhl *et al*.) that demonstrated improvement in psoriatic skin lesions when PRP was applied to the skin through multiple delivery methods (*i.e*., intradermal, subdermal, subcutaneous). Nevertheless, none of these studies mentioned patients with psoriatic arthritis [47-49].

## CONCLUSION

Given the safety profile of PRP in autoimmune disorders and the increased use in related musculoskeletal conditions, such as OA, there is a need for further research to investigate potential avenues for PRP treatment. Of note, the role of PRP in OA has been widely evaluated and was excluded from this report as the abundance of literature merits its own separate review to fully detail the outcomes in this patient population. Focused research on molecular mechanisms of platelet interaction could fill the knowledge gaps and improve our understanding of the exact role PRP has in improving outcomes in autoimmune conditions.

## LIMITATIONS

The main limitations of our review include a lack of high-quality randomized, double-blind placebo-controlled trials, a lack of standard PRP formulations, differences in treatment strategies, and short follow-up periods. Many of the sources included in this review include case reports, which make it hard to draw large-scale conclusions about the effectiveness of PRP use. Moreover, some of the trials available include a small number of patients, which limits the power and generalizability of the results. Additionally, varying treatment strategies (numbers of injections) in the studies may impact results either positively or negatively; however, their effect is unclear. Finally, different PRP formulations were used in the studies. The preparations differ in the commercial kit being utilized for the separation of blood components, the number of times being centrifuged, as well as the rate and time interval, activation substance, and anticoagulant used. Not only that, but different studies either mention leukocyte-rich or leukocyte-poor PRP or do not mention leukocyte content at all, which is another variable. This makes it difficult to compare results as the products are not the same across reports. The process of preparing PRP leaves questions about how different preparations may yield different results. Given all of these limitations, it is unfortunately too soon to conclude if PRP is a real treatment option for RD. Further higher-quality studies are required to make legitimate recommendations.

## Figures and Tables

**Fig. (2):**
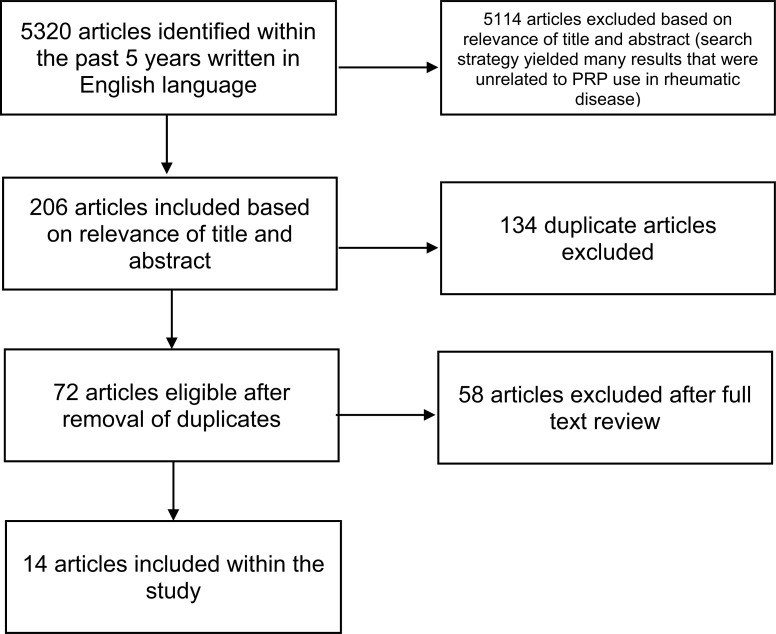
Flow sheet demonstrating number of records excluded by each exclusion criterion.

**Table 1:** Resources included in literature search organized by disease entity.

***Rheumatoid Arthritis***	Evaluating the Efficacy of Intra-articular Injections of Platelet Rich Plasma (PRP) in Rheumatoid Arthritis Patients and its Impact on Inflammatory Cytokines, Disease Activity and Quality of Life.	Platelet-Rich Plasma for Rheumatoid Arthritis: A Case Series.	Platelet Rich Plasma for Treatment of Rheumatoid Arthritis: Case Series and Review of Literature.
***Systemic Sclerosis***	Combined platelet-rich plasma and lipofilling treatment provides great improvement in facial skin-induced lesion regeneration for scleroderma patients.	-	-
***Sjogren's Syndrome***	Randomised, prospective clinical trial of platelet-rich plasma injection in the management of severe dry eye.	Homologous platelet-rich plasma for the treatment of knee involvement in primary Sjögrens syndrome.	-
***Vasculitis***	Autologous platelet-rich plasma in the treatment of refractory wounds in cutaneous leukocytoclastic vasculitis complicated with hypertension (grade 2 moderate risk): A case report.	Autologous platelet rich plasma in the management of non-healing vasculitic ulcers.	Characterization of autologous platelet rich plasma (PRP) and its biological effects in patients with Behcet’s Disease.
***Sarcoidosis***	Platelet-Rich Plasma Injection and Cutaneous Sarcoidal Granulomas.	Platelet-rich plasma injections and the development of cutaneous sarcoid lesions: A case report.	-
***Crystalline arthropathy***	Flare of Gout from Injection of Platelet-Rich Plasma.	Pseudogout flare after platelet-rich plasma injection: A case series.	-
***Discoid Lupus Erythematosus***	Platelet rich plasma for the treatment of scarring alopecia due to discoid lupus erythematosus	-	-

## LIST OF ABBREVIATIONS

BD: Behcet’s Disease
DAS 28: Disease Activity Score 28
DMARDS: Disease-Modifying Antirheumatic Drugs
EGF: Epidermal Growth Factor
FGF: Fibroblast Growth Factor
HA: Hyaluronic Acid
HAQ: Health Assessment Questionnaire
MCP: Metacarpophalangeal
MCTD: Mixed Connective Tissue Disease
PAS-II: Patient Activity Scale II
PDGF: Platelet-Derived Growth Factor
PIP: Proximal Interphalangeal
PRP: Platelet-Rich Plasma
RA: Rheumatoid Arthritis
RCT: Randomized Controlled Trial
RD: Rheumatic Diseases
SLE: Systemic Lupus Erythematosus
SS: Sjogren’s Syndrome
TGF-β: Transforming Growth Factor-β
VAS: Visual Analog Scale
VEGF: Vascular Endothelial Growth Factor
