# Supplementary material for: Current State of Platelet-rich Plasma in the Treatment of Rheumatic Disease: A Retrospective Review of the Literature
Source: Curr Rheumatol Rev. 2023 Aug 3;19(4):400–7. doi: 10.2174/1573397119666230420112017 (PMC10523354; doi:10.2174/1573397119666230420112017)
Supplement: Supplementary file 1 — Supplementary material is available on the publisher’s website along with the published article. [file CRR-19-400_SD1.pdf]

Supplementary Material

Current State of Platelet-rich Plasma in the Treatment of Rheumatic Disease: A Retrospective Review of the Literature

Adam Jacobs[1], Omar Elghawy[2], Diego Lugo Baruqui[1] and Ahmed Aly Elghawy[3*]

[1]*Mount Sinai Medical Center, Miami Beach, FL, United States;* [2]*University of Virginia School of Medicine, Charlottesville, VA, United States;* [3]*Department of Rheumatologic and Immunologic Disease, Cleveland Clinic, Cleveland, OH, United States*

Search strategy used in PubMed database

Search Topic	**#**	**Search Terms**
Population:<br><br>*Human or animal subjects of Psoriatic Arthritis, In vitro cell lines replicating Psoriatic Arthritis*	#1	"(Rheumatoid Arthritis"[Mesh]) OR (Rheumatoid [All Fields])<br><br>OR (RA[All Fields]) OR (Rheumato*[All Fields])
	#2	("joints"[MeSH Terms] OR "joints"[All Fields] OR "arthritis"[All Fields]) AND ("rheumatoid"[All fields]
	#3	(inflammatory[All Fields]) AND (arthritis[All Fields]) AND ((cell lines[All Fields]) OR (in vitro [All Fields]))
	#4	#1 OR #2 OR #3
Intervention:<br><br>*Platelet-rich plasma, Injection*	#5	" Platelet-rich plasma"OR "PRP"
	#6	" Platelet-rich plasma "[Mesh] OR "Injections"[Mesh] OR<br><br>intraarticul*
	#7	#5 OR #6
	#8	#4 AND #7
Study design:<br><br>*RCTs, cohort studies, case control studies, case series*	#9	("clinical trial"[All Fields] OR "clinical trials as topic"[MeSH Terms] OR "clinical trials"[All Fields]OR “case series”[All Fields] OR “"randomized controlled trial"[All Fields] OR "randomized controlled trials as topic"[MeSH Terms] OR "randomized controlled trial"[pt] OR "controlled clinical trial"[pt] OR "randomized"[tiab] OR "randomly"[tiab] OR "trial"[ti] OR "randomised controlled trial"[All Fields] OR "randomized controlled trial"[pt] "prospective studies"[MeSH Terms] OR "prospective studies"[All Fields] OR "retrospective studies"[MeSH Terms] OR "retrospective studies"[All Fields] OR "retrospective study"[All Fields]
	#10	#8 AND #9

1875-6360/23 $65.00+.00 © 2023 Bentham Science Publishers

	#11	(“biography”[Publication Type] OR “comment”[Publication Type] OR “directory”[Publication Type] OR “editorial”[Publication Type] OR “festschrift”[Publication Type] OR “interview”[Publication Type] OR “lecture”[Publication Type] OR “legal case”[Publication Type] OR “legislation”[Publication Type] OR “letter”[Publication Type] OR “news”[Publication Type] OR “newspaper article”[Publication Type] OR “patient education handout”[Publication Type] OR “popular work”[Publication Type] OR “congress”[Publication Type] OR “consensus development conference”[Publication Type] OR “consensus development conference, nih”[Publication Type] OR “practice guideline”[Publication Type]) OR “Review”[Publication Type])
	#12	#10 NOT #11

Search Topic	**#**	**Search Terms**
	#1	Platelet Rich Plasma in Rheumatoid Arthritis

Search Topic	**#**	**Search Terms**
Population: *Human or animal subjects of Rheumatoid Arthritis, In vitro cell lines replicating Rheumatoid Arthritis*	#1	"(Psoriatic Arthritis"[Mesh]) OR (Psoriasis [All Fields]) OR (PsA[All Fields]) OR (Psoria*[All Fields])
	#2	("joints"[MeSH Terms] OR "joints"[All Fields] OR "arthritis"[All Fields]) AND ("psoriatic"[All fields]
	#3	(inflammatory[All Fields]) AND (arthritis[All Fields]) AND ((cell lines[All Fields]) OR (in vitro [All Fields]))
	#4	#1 OR #2 OR #3
Intervention: *Platelet-rich plasma, Injection*	#5	" Platelet-rich plasma"OR "PRP"
	#6	" Platelet-rich plasma "[Mesh] OR "Injections"[Mesh] OR intraarticul*
	#7	#5 OR #6
	#8	#4 AND #7
Study design: *RCTs, cohort studies, case control studies, case series*	#9	("clinical trial"[All Fields] OR "clinical trials as topic"[MeSH Terms] OR "clinical trials"[All Fields]OR “case series”[All Fields] OR “"randomized controlled trial"[All Fields] OR "randomized controlled trials as topic"[MeSH Terms] OR "randomized controlled trial"[pt] OR "controlled clinical trial"[pt] OR "randomized"[tiab] OR "randomly"[tiab] OR

		"trial"[ti] OR "randomised controlled trial"[All Fields] OR "randomized controlled trial"[pt] "prospective studies"[MeSH Terms] OR "prospective studies"[All Fields] OR "retrospective studies"[MeSH Terms] OR "retrospective studies"[All Fields] OR "retrospective study"[All Fields]
	#10	#8 AND #9
	#11	(“biography”[Publication Type] OR “comment”[Publication Type] OR “directory”[Publication Type] OR “editorial”[Publication Type] OR “festschrift”[Publication Type] OR “interview”[Publication Type] OR “lecture”[Publication Type] OR “legal case”[Publication Type] OR “legislation”[Publication Type] OR “letter”[Publication Type] OR “news”[Publication Type] OR “newspaper article”[Publication Type] OR “patient education handout”[Publication Type] OR “popular work”[Publication Type] OR “congress”[Publication Type] OR “consensus development conference”[Publication Type] OR “consensus development conference, nih”[Publication Type] OR “practice guideline”[Publication Type]) OR “Review”[Publication Type])
	#12	#10 NOT #11

Search Topic	**#**	**Search Terms**
	#1	Platelet Rich Plasma in Psoriasis

Search Topic	**#**	**Search Terms**
Population: *Human or animal subjects of Rheumatoid Arthritis, In vitro cell lines replicating Rheumatoid Arthritis*	#1	"(Reactive Arthritis"[Mesh]) OR (Reactive Arthritis [All Fields]) OR (Rheumato*[All Fields])
	#2	("joints"[MeSH Terms] OR "joints"[All Fields] OR "arthritis"[All Fields]) AND ("reactive"[All fields]
	#3	(inflammatory[All Fields]) AND (arthritis[All Fields]) AND ((cell lines[All Fields]) OR (in vitro [All Fields]))
	#4	#1 OR #2 OR #3
Intervention: *Platelet-rich plasma, Injection*	#5	" Platelet-rich plasma"OR "PRP"
	#6	" Platelet-rich plasma "[Mesh] OR "Injections"[Mesh] OR intraarticul*
	#7	#5 OR #6

	#8	#4 AND #7
Study design: *RCTs, cohort studies, case control studies, case series*	#9	("clinical trial"[All Fields] OR "clinical trials as topic"[MeSH Terms] OR "clinical trials"[All Fields]OR “case series”[All Fields] OR “"randomized controlled trial"[All Fields] OR "randomized controlled trials as topic"[MeSH Terms] OR "randomized controlled trial"[pt] OR "controlled clinical trial"[pt] OR "randomized"[tiab] OR "randomly"[tiab] OR "trial"[ti] OR "randomised controlled trial"[All Fields] OR "randomized controlled trial"[pt] "prospective studies"[MeSH Terms] OR "prospective studies"[All Fields] OR "retrospective studies"[MeSH Terms] OR "retrospective studies"[All Fields] OR "retrospective study"[All Fields]
	#10	#8 AND #9
	#11	(“biography”[Publication Type] OR “comment”[Publication Type] OR “directory”[Publication Type] OR “editorial”[Publication Type] OR “festschrift”[Publication Type] OR “interview”[Publication Type] OR “lecture”[Publication Type] OR “legal case”[Publication Type] OR “legislation”[Publication Type] OR “letter”[Publication Type] OR “news”[Publication Type] OR “newspaper article”[Publication Type] OR “patient education handout”[Publication Type] OR “popular work”[Publication Type] OR “congress”[Publication Type] OR “consensus development conference”[Publication Type] OR “consensus development conference, nih”[Publication Type] OR “practice guideline”[Publication Type]) OR “Review”[Publication Type])
	#12	#10 NOT #11

Search Topic	**#**	**Search Terms**
	#1	Platelet Rich Plasma in Reactive Arthritis

Search Topic	**#**	**Search Terms**
Population: *Human or animal subjects of Rheumatoid Arthritis, In vitro cell lines replicating Rheumatoid Arthritis*	#1	"(Gout"[Mesh]) OR (Gout [All Fields])
	#2	("joints"[MeSH Terms] OR "joints"[All Fields] OR "arthritis"[All Fields]) AND ("gout"[All fields]
	#3	(inflammatory[All Fields]) AND (gout [All Fields]) AND ((cell lines[All Fields]) OR (in vitro [All Fields]))
	#4	#1 OR #2 OR #3
Intervention:	#5	" Platelet-rich plasma"OR "PRP"

Platelet-rich plasma, Injection		
	#6	" Platelet-rich plasma "[Mesh] OR "Injections"[Mesh] OR<br><br>intraarticul*
	#7	#5 OR #6
	#8	#4 AND #7
Study design:<br><br>*RCTs, cohort studies, case control studies, case series*	#9	("clinical trial"[All Fields] OR "clinical trials as topic"[MeSH Terms] OR "clinical trials"[All Fields]OR "case series"[All Fields] OR ""randomized controlled trial"[All Fields] OR "randomized controlled trials as topic"[MeSH Terms] OR "randomized controlled trial"[pt] OR "controlled clinical trial"[pt] OR "randomized"[tiab] OR "randomly"[tiab] OR "trial"[ti] OR "randomised controlled trial"[All Fields] OR "randomized controlled trial"[pt] "prospective studies"[MeSH Terms] OR "prospective studies"[All Fields] OR "retrospective studies"[MeSH Terms] OR "retrospective studies"[All Fields] OR "retrospective study"[All Fields]
	#10	#8 AND #9
	#11	("biography"[Publication Type] OR "comment"[Publication Type] OR "directory"[Publication<br><br>Type] OR "editorial"[Publication Type] OR "festschrift"[Publication<br><br>Type] OR "interview"[Publication Type] OR "lecture"[Publication<br><br>Type] OR "legal case"[Publication Type] OR "legislation"[Publication<br><br>Type] OR "letter"[Publication Type] OR "news"[Publication Type] OR<br><br>"newspaper article"[Publication Type] OR "patient education<br><br>handout"[Publication Type] OR "popular work"[Publication Type] OR<br><br>"congress"[Publication Type] OR "consensus development<br><br>conference"[Publication Type] OR "consensus development<br><br>conference, nih"[Publication Type] OR "practice<br><br>guideline"[Publication Type]) OR "Review"[Publication Type])
	#12	#10 NOT #11

Search Topic	**#**	**Search Terms**
	#1	Platelet Rich Plasma in Gout

Search Topic	**#**	**Search Terms**
Population:<br><br>*Human or animal subjects of Rheumatoid Arthritis, In vitro cell lines replicating Rheumatoid Arthritis*	#1	"(Vasculitis "[Mesh]) OR (Vasculitis [All Fields])<br><br>OR (Giant cell arteritis [All Fields]) OR (Polyangiitis [All Fields])
	#2	("joints"[MeSH Terms] OR "joints"[All Fields] OR "vasculitis"[All Fields]) AND ("arteritis"[All fields]
	#3	(inflammatory[All Fields]) AND (vasculitis[All Fields]) AND ((cell lines[All Fields]) OR (in

		vitro [All Fields]))
	#4	#1 OR #2 OR #3
Intervention: *Platelet-rich plasma, Injection*	#5	" Platelet-rich plasma"OR "PRP"
	#6	" Platelet-rich plasma "[Mesh] OR "Injections"[Mesh] OR intraarticul*
	#7	#5 OR #6
	#8	#4 AND #7
Study design: *RCTs, cohort studies, case control studies, case series*	#9	("clinical trial"[All Fields] OR "clinical trials as topic"[MeSH Terms] OR "clinical trials"[All Fields]OR “case series”[All Fields] OR “"randomized controlled trial"[All Fields] OR "randomized controlled trials as topic"[MeSH Terms] OR "randomized controlled trial"[pt] OR "controlled clinical trial"[pt] OR "randomized"[tiab] OR "randomly"[tiab] OR "trial"[ti] OR "randomised controlled trial"[All Fields] OR "randomized controlled trial"[pt] "prospective studies"[MeSH Terms] OR "prospective studies"[All Fields] OR "retrospective studies"[MeSH Terms] OR "retrospective studies"[All Fields] OR "retrospective study"[All Fields]
	#10	#8 AND #9
	#11	(“biography”[Publication Type] OR “comment”[Publication Type] OR “directory”[Publication Type] OR “editorial”[Publication Type] OR “festschrift”[Publication Type] OR “interview”[Publication Type] OR “lecture”[Publication Type] OR “legal case”[Publication Type] OR “legislation”[Publication Type] OR “letter”[Publication Type] OR “news”[Publication Type] OR “newspaper article”[Publication Type] OR “patient education handout”[Publication Type] OR “popular work”[Publication Type] OR “congress”[Publication Type] OR “consensus development conference”[Publication Type] OR “consensus development conference, nih”[Publication Type] OR “practice guideline”[Publication Type]) OR “Review”[Publication Type])
	#12	#10 NOT #11

Search Topic	**#**	**Search Terms**
	#1	Platelet Rich Plasma in Vasculitis

Search Topic	**#**	**Search Terms**
Population: *Human or animal subjects of Rheumatoid Arthritis, In vitro cell lines replicating*	#1	"(Inflammatory myopathy"[Mesh]) OR (myositis [All Fields]) OR (polymyositis[All Fields]) OR (dermatomyositis[All Fields])

Rheumatoid Arthritis		
	#2	("joints"[MeSH Terms] OR "joints"[All Fields]
	#3	(inflammatory[All Fields]) AND (myositis[All Fields]) AND ((cell lines[All Fields]) OR (in vitro [All Fields]))
	#4	#1 OR #2 OR #3
Intervention: *Platelet-rich plasma, Injection*	#5	" Platelet-rich plasma"OR "PRP"
	#6	" Platelet-rich plasma "[Mesh] OR "Injections"[Mesh] OR intraarticul*
	#7	#5 OR #6
	#8	#4 AND #7
Study design: *RCTs, cohort studies, case control studies, case series*	#9	("clinical trial"[All Fields] OR "clinical trials as topic"[MeSH Terms] OR "clinical trials"[All Fields]OR “case series”[All Fields] OR “"randomized controlled trial"[All Fields] OR "randomized controlled trials as topic"[MeSH Terms] OR "randomized controlled trial"[pt] OR "controlled clinical trial"[pt] OR "randomized"[tiab] OR "randomly"[tiab] OR "trial"[ti] OR "randomised controlled trial"[All Fields] OR "randomized controlled trial"[pt] "prospective studies"[MeSH Terms] OR "prospective studies"[All Fields] OR "retrospective studies"[MeSH Terms] OR "retrospective studies"[All Fields] OR "retrospective study"[All Fields]
	#10	#8 AND #9
	#11	(“biography”[Publication Type] OR “comment”[Publication Type] OR “directory”[Publication Type] OR “editorial”[Publication Type] OR “festschrift”[Publication Type] OR “interview”[Publication Type] OR “lecture”[Publication Type] OR “legal case”[Publication Type] OR “legislation”[Publication Type] OR “letter”[Publication Type] OR “news”[Publication Type] OR “newspaper article”[Publication Type] OR “patient education handout”[Publication Type] OR “popular work”[Publication Type] OR “congress”[Publication Type] OR “consensus development conference”[Publication Type] OR “consensus development conference, nih”[Publication Type] OR “practice guideline”[Publication Type]) OR “Review”[Publication Type])
	#12	#10 NOT #11

Search Topic	**#**	**Search Terms**
	#1	Platelet Rich Plasma in Myositis

Search Topic	#	Search Terms
Population:<br><br>*Human or animal subjects of Rheumatoid Arthritis, In vitro cell lines replicating Rheumatoid Arthritis*	#1	"(Lupus"[Mesh]) OR (Lupus [All Fields])<br><br>OR (SLE[All Fields]) OR (Systemic Lupus Erythematosus[All Fields])
	#2	("joints"[MeSH Terms] OR "joints"[All Fields] OR "lupus"[All Fields]) AND ("arteritis"[All fields]
	#3	(inflammatory[All Fields]) AND (lupus[All Fields]) AND ((cell lines[All Fields]) OR (in vitro [All Fields]))
	#4	#1 OR #2 OR #3
Intervention:<br><br>*Platelet-rich plasma, Injection*	#5	" Platelet-rich plasma"OR "PRP"
	#6	" Platelet-rich plasma "[Mesh] OR "Injections"[Mesh] OR<br><br>intraarticul*
	#7	#5 OR #6
	#8	#4 AND #7
Study design:<br><br>*RCTs, cohort studies, case control studies, case series*	#9	("clinical trial"[All Fields] OR "clinical trials as topic"[MeSH Terms] OR "clinical trials"[All Fields]OR “case series”[All Fields] OR “"randomized controlled trial"[All Fields] OR "randomized controlled trials as topic"[MeSH Terms] OR "randomized controlled trial"[pt] OR "controlled clinical trial"[pt] OR "randomized"[tiab] OR "randomly"[tiab] OR "trial"[ti] OR "randomised controlled trial"[All Fields] OR "randomized controlled trial"[pt] "prospective studies"[MeSH Terms] OR "prospective studies"[All Fields] OR "retrospective studies"[MeSH Terms] OR "retrospective studies"[All Fields] OR "retrospective study"[All Fields]
	#10	#8 AND #9
	#11	(“biography”[Publication Type] OR “comment”[Publication Type] OR “directory”[Publication<br><br>Type] OR “editorial”[Publication Type] OR “festschrift”[Publication<br><br>Type] OR “interview”[Publication Type] OR “lecture”[Publication<br><br>Type] OR “legal case”[Publication Type] OR “legislation”[Publication<br><br>Type] OR “letter”[Publication Type] OR “news”[Publication Type] OR<br><br>“newspaper article”[Publication Type] OR “patient education<br><br>handout”[Publication Type] OR “popular work”[Publication Type] OR<br><br>“congress”[Publication Type] OR “consensus development<br><br>conference”[Publication Type] OR “consensus development<br><br>conference, nih”[Publication Type] OR “practice<br><br>guideline”[Publication Type]) OR “Review”[Publication Type])
	#12	#10 NOT #11

Search Topic	**#**	**Search Terms**
	#1	Platelet Rich Plasma in Lupus

Search Topic	**#**	**Search Terms**
Population: <br>*Human or animal subjects of Rheumatoid Arthritis, In vitro cell lines replicating Rheumatoid Arthritis*	#1	"(Ankylosing spondylitis"[Mesh]) OR (Ankylosing spondylitis [All Fields]) <br>OR (AS[All Fields])
	#2	("joints"[MeSH Terms] OR "joints"[All Fields] OR "Ankylosing spondylitis"[All Fields]) AND ("inflammatory"[All fields]
	#3	(inflammatory[All Fields]) AND (Ankylosing spondylitis [All Fields]) AND ((cell lines[All Fields]) OR (in vitro [All Fields]))
	#4	#1 OR #2 OR #3
Intervention: <br>*Platelet-rich plasma, Injection*	#5	" Platelet-rich plasma"OR "PRP"
	#6	" Platelet-rich plasma "[Mesh] OR "Injections"[Mesh] OR <br>intraarticul*
	#7	#5 OR #6
	#8	#4 AND #7
Study design: <br>*RCTs, cohort studies, case control studies, case series*	#9	("clinical trial"[All Fields] OR "clinical trials as topic"[MeSH Terms] OR "clinical trials"[All Fields]OR "case series"[All Fields] OR ""randomized controlled trial"[All Fields] OR "randomized controlled trials as topic"[MeSH Terms] OR "randomized controlled trial"[pt] OR "controlled clinical trial"[pt] OR "randomized"[tiab] OR "randomly"[tiab] OR "trial"[ti] OR "randomised controlled trial"[All Fields] OR "randomized controlled trial"[pt] "prospective studies"[MeSH Terms] OR "prospective studies"[All Fields] OR "retrospective studies"[MeSH Terms] OR "retrospective studies"[All Fields] OR "retrospective study"[All Fields]
	#10	#8 AND #9
	#11	("biography"[Publication Type] OR "comment"[Publication Type] OR "directory"[Publication <br>Type] OR "editorial"[Publication Type] OR "festschrift"[Publication <br>Type] OR "interview"[Publication Type] OR "lecture"[Publication <br>Type] OR "legal case"[Publication Type] OR "legislation"[Publication <br>Type] OR "letter"[Publication Type] OR "news"[Publication Type] OR <br>"newspaper article"[Publication Type] OR "patient education <br>handout"[Publication Type] OR "popular work"[Publication Type] OR <br>"congress"[Publication Type] OR "consensus development <br>conference"[Publication Type] OR "consensus development <br>conference, nih"[Publication Type] OR "practice

		guideline"[Publication Type]) OR "Review"[Publication Type])
	#12	#10 NOT #11

Search Topic	**#**	**Search Terms**
	#1	Platelet Rich Plasma in Ankylosing Spondylitis

Search Topic	**#**	**Search Terms**
Population: *Human or animal subjects of Rheumatoid Arthritis, In vitro cell lines replicating Rheumatoid Arthritis*	#1	"(Scleroderma"[Mesh]) OR (Scleroderma [All Fields]) OR (Crest Syndrome[All Fields])
	#2	("joints"[MeSH Terms] OR "joints"[All Fields] OR "ascleroderma"[All Fields]) AND ("inflammatory"[All fields]
	#3	(inflammatory[All Fields]) AND (skin[All Fields]) AND ((cell lines[All Fields]) OR (in vitro [All Fields]))
	#4	#1 OR #2 OR #3
Intervention: *Platelet-rich plasma, Injection*	#5	" Platelet-rich plasma"OR "PRP"
	#6	" Platelet-rich plasma "[Mesh] OR "Injections"[Mesh] OR intraarticul*
	#7	#5 OR #6
	#8	#4 AND #7
Study design: *RCTs, cohort studies, case control studies, case series*	#9	("clinical trial"[All Fields] OR "clinical trials as topic"[MeSH Terms] OR "clinical trials"[All Fields]OR "case series"[All Fields] OR ""randomized controlled trial"[All Fields] OR "randomized controlled trials as topic"[MeSH Terms] OR "randomized controlled trial"[pt] OR "controlled clinical trial"[pt] OR "randomized"[tiab] OR "randomly"[tiab] OR "trial"[ti] OR "randomised controlled trial"[All Fields] OR "randomized controlled trial"[pt] "prospective studies"[MeSH Terms] OR "prospective studies"[All Fields] OR "retrospective studies"[MeSH Terms] OR "retrospective studies"[All Fields] OR "retrospective study"[All Fields]
	#10	#8 AND #9
	#11	("biography"[Publication Type] OR "comment"[Publication Type] OR "directory"[Publication Type] OR "editorial"[Publication Type] OR "festschrift"[Publication Type] OR "interview"[Publication Type] OR "lecture"[Publication Type] OR "legal case"[Publication Type] OR "legislation"[Publication Type] OR "letter"[Publication Type] OR "news"[Publication Type] OR "newspaper article"[Publication Type] OR "patient education

		handout"[Publication Type] OR "popular work"[Publication Type] OR "congress"[Publication Type] OR "consensus development conference"[Publication Type] OR "consensus development conference, nih"[Publication Type] OR "practice guideline"[Publication Type]) OR "Review"[Publication Type])
	#12	#10 NOT #11

Search Topic	**#**	**Search Terms**
	#1	Platelet Rich Plasma in Scleroderma

Search Topic	**#**	**Search Terms**
Population: *Human or animal subjects of Rheumatoid Arthritis, In vitro cell lines replicating Rheumatoid Arthritis*	#1	"(Sarcoidosis"[Mesh]) OR (Sarcoid [All Fields]) OR (Sarcoidosis[All Fields])
	#2	("joints"[MeSH Terms] OR "joints"[All Fields] OR "scleroderma"[All Fields]) AND ("inflammatory"[All fields]
	#3	(inflammatory[All Fields]) AND (Sarcoid[All Fields]) AND ((cell lines[All Fields]) OR (in vitro [All Fields]))
	#4	#1 OR #2 OR #3
Intervention: *Platelet-rich plasma, Injection*	#5	" Platelet-rich plasma"OR "PRP"
	#6	" Platelet-rich plasma "[Mesh] OR "Injections"[Mesh] OR intraarticul*
	#7	#5 OR #6
	#8	#4 AND #7
Study design: *RCTs, cohort studies, case control studies, case series*	#9	("clinical trial"[All Fields] OR "clinical trials as topic"[MeSH Terms] OR "clinical trials"[All Fields]OR "case series"[All Fields] OR ""randomized controlled trial"[All Fields] OR "randomized controlled trials as topic"[MeSH Terms] OR "randomized controlled trial"[pt] OR "controlled clinical trial"[pt] OR "randomized"[tiab] OR "randomly"[tiab] OR "trial"[ti] OR "randomised controlled trial"[All Fields] OR "randomized controlled trial"[pt] "prospective studies"[MeSH Terms] OR "prospective studies"[All Fields] OR "retrospective studies"[MeSH Terms] OR "retrospective studies"[All Fields] OR "retrospective study"[All Fields]
	#10	#8 AND #9
	#11	("biography"[Publication Type] OR "comment"[Publication Type] OR "directory"[Publication Type] OR "editorial"[Publication Type] OR "festschrift"[Publication

		Type] OR “interview”[Publication Type] OR “lecture”[Publication Type] OR “legal case”[Publication Type] OR “legislation”[Publication Type] OR “letter”[Publication Type] OR “news”[Publication Type] OR “newspaper article”[Publication Type] OR “patient education handout”[Publication Type] OR “popular work”[Publication Type] OR “congress”[Publication Type] OR “consensus development conference”[Publication Type] OR “consensus development conference, nih”[Publication Type] OR “practice guideline”[Publication Type]) OR “Review”[Publication Type])
	#12	#10 NOT #11

Search Topic	**#**	**Search Terms**
	#1	Platelet Rich Plasma in Sarcoid

Search Topic	**#**	**Search Terms**
Population: *Human or animal subjects of Rheumatoid Arthritis, In vitro cell lines replicating Rheumatoid Arthritis*	#1	"(IgG4"[Mesh]) OR (IgG4-related disease [All Fields]) OR (IgG4-RD[All Fields]) OR (IgG4[All Fields])
	#2	("joints"[MeSH Terms] OR "joints"[All Fields] OR "IgG4"[All Fields]) AND ("joint"[All fields]
	#3	(inflammatory[All Fields]) AND (IgG4[All Fields]) AND ((cell lines[All Fields]) OR (in vitro [All Fields]))
	#4	#1 OR #2 OR #3
Intervention: *Platelet-rich plasma, Injection*	#5	" Platelet-rich plasma"OR "PRP"
	#6	" Platelet-rich plasma "[Mesh] OR "Injections"[Mesh] OR intraarticul*
	#7	#5 OR #6
	#8	#4 AND #7
Study design: *RCTs, cohort studies, case control studies, case series*	#9	("clinical trial"[All Fields] OR "clinical trials as topic"[MeSH Terms] OR "clinical trials"[All Fields]OR “case series”[All Fields] OR “"randomized controlled trial"[All Fields] OR "randomized controlled trials as topic"[MeSH Terms] OR "randomized controlled trial"[pt] OR "controlled clinical trial"[pt] OR "randomized"[tiab] OR "randomly"[tiab] OR "trial"[ti] OR "randomised controlled trial"[All Fields] OR "randomized controlled trial"[pt] "prospective studies"[MeSH Terms] OR "prospective studies"[All Fields] OR "retrospective studies"[MeSH Terms] OR "retrospective studies"[All Fields] OR "retrospective study"[All Fields]

	#10	#8 AND #9
	#11	(“biography”[Publication Type] OR “comment”[Publication Type] OR “directory”[Publication Type] OR “editorial”[Publication Type] OR “festschrift”[Publication Type] OR “interview”[Publication Type] OR “lecture”[Publication Type] OR “legal case”[Publication Type] OR “legislation”[Publication Type] OR “letter”[Publication Type] OR “news”[Publication Type] OR “newspaper article”[Publication Type] OR “patient education handout”[Publication Type] OR “popular work”[Publication Type] OR “congress”[Publication Type] OR “consensus development conference”[Publication Type] OR “consensus development conference, nih”[Publication Type] OR “practice guideline”[Publication Type]) OR “Review”[Publication Type])
	#12	#10 NOT #11

Search Topic	**#**	**Search Terms**
	#1	Platelet Rich Plasma in IgG4-Related Disease

DISCLAIMER: The above article has been published, as is, ahead-of-print, to provide early visibility but is not the final version. Major publication processes like copyediting, proofing, typesetting and further review are still to be done and may lead to changes in the final published version, if it is eventually published. All legal disclaimers that apply to the final published article also apply to this ahead-of-print version.
